# A molecular dynamics study of the sputtering processes of beryllium species by hydrogenic plasma

**DOI:** 10.1038/s41598-025-98065-1

**Published:** 2025-04-29

**Authors:** Alexander Liptak, Kerry D. Lawson, Mohammad I. Hasan

**Affiliations:** 1 Diamond Light Source Ltd, Harwell Science and Innovation Campus, Didcot, OX11 0DE UK; 2 Department of Electrical Engineering and Electronics, University of Liverpool, Liverpool, L69 3GJ UK; 3 UKAEA (UK Atomic Energy Authority), Culham Campus, Abingdon, OX14 3DB UK

**Keywords:** Magnetically confined plasmas, Atomistic models, Metals and alloys

## Abstract

The plasma-facing components of nuclear fusion reactors are continuously subjected to high thermal loads and bombardment by hydrogenic ions, resulting in erosion of the first-wall material and sputtering of atomic and molecular species into the edge plasma. The management of reactor component lifetimes and control of sputtered plasma impurities remain open problems in fusion research, where quantifying the sputtering yields and understanding the sputtering processes of each impurity species is pivotal for planning an effective maintenance schedule and ensuring optimal plasma performance. Although the sputtering of atomic species is historically well-established, the sputtering of molecules from metallic first-wall materials such as beryllium has only recently been confirmed possible and relevant for fusion reactors. In this study, a molecular dynamics model is used to investigate the sputtering of atomic beryllium, Be$_2$ dimers, Be$_3$ trimers, beryllium hydrides, and hydrogenic dimers. The sputtering yield of each species is reported, and the observed ion trajectory preferential sputtering behaviour is presented; beryllium lattice thermal effects as well as bombarding ion isotope effects are discussed where relevant. A theoretical description of the universal sputtering yield relation and its various corrections are explored in detail, and the molecular dynamics model is benchmarked against analytical, computational and experimental data.

## Introduction

Beryllium, a low-Z metal with excellent thermal and mechanical properties, is a material with well-established aerospace and nuclear applications. Despite its toxicity, it is regularly considered the leading candidate for plasma-facing components (PFCs) in the context of nuclear fusion^1,2^, having been proposed as the in-vessel material for the International Thermonuclear Experimental Reactor (ITER)^3^ and implemented for testing as part of the Joint European Torus ITER-like Wall (JET-ILW) experiment^4^. Although ITER has recently announced its intention to move away from beryllium and instead use tungsten as the first wall material, beryllium continues to be a fusion-relevant material due to its good thermal and mechanical properties, in-situ repairability^2^, its low atomic number which results in minimal contributions to plasma radiation losses when considered as a plasma impurity, as well as excellent neutron multiplication performance necessary for breeding tritium fuel^5^.

During the operation of conventional fusion reactors, PFCs are continuously bombarded by high-temperature hydrogenic plasma species, resulting in the ion-assisted removal process (sputtering) of beryllium atoms, or as recently confirmed to be possible^6^ and relevant^7^, beryllium hydride molecules. Understanding the sputtering processes and quantifying the sputtering yields of all relevant beryllium compounds is vital for assessing the multidisciplinary implications of PFC erosion; from an engineering perspective, erosion rates indicate the expected lifetime and necessary maintenance schedule of a reactor’s first-wall components, while the resulting formation of beryllium dust poses a health hazard that requires strict monitoring. Additionally, sputtering is considered an uncontrolled source of plasma impurities^8^; while useful in controlled circumstances for reducing divertor heat loads, such impurities can also impact the performance of a reactor by diluting the plasma and radiating away heat if not adequately managed. As such, modelling plasma impurity generation and transport remains an important topic in nuclear fusion^9^.

Although well-established methods exist for estimating atomic sputtering yields from solid targets by highly energetic ions, such as the binary collision approximation (BCA)^10^ and its popular software implementation SRIM^11^, these techniques are often inaccurate for modelling collisions with kinetic energies below $\sim$ 100 eV, where lattice forces and many-body interactions play a more significant role^12^; this plasma-surface interaction energy range is, however, of particular interest to both the nuclear fusion^13,14^ and plasma processing^15,16^ industries, where the use of molecular dynamics (MD) is often preferred^17,18^. In addition, MD models can be used to study increasingly complex and realistic scenarios^19^, such as the sputtering of molecular species from irradiated samples with trapped impurities, at the expense of increased computational cost. The usefulness of MD in the context of nuclear fusion is also highlighted by the recent development of many new interatomic potentials purpose-built for the study of PFCs^20,21^.

In this work, we utilise a previously described MD model of a rough beryllium surface^22^ to study the sputtering processes of beryllium species by deuterium and tritium ions with energies up to 150 eV. The sputtering of atomic Be, as well as the BeH and Be$_2$ dimers (and the Be$_3$ trimer with an extended bombardment ion energy range), are discussed. The sputtering yield as a function of bombarding ion energy is reported in the Results section in the form of fitted parameters to the Bohdansky universal sputtering relation^23^, as common with existing sputtering yield studies^24^; for the dimers and trimers, this sputtering dependence on ion energy is described on a case-by-case basis. Furthermore, as the sputtering yield of atomic beryllium was previously identified to exhibit a weak dependence on the PFC surface temperature at low bombarding ion energies^25^, this relationship is also investigated here using multiple MD systems with varying beryllium surface temperatures up to 1100 K.

The significance of the reported results is discussed in the following subsection, which also includes a benchmark of the MD model against previously reported experimental and modelling results. Further details regarding the preparation and validation of the MD system and the counting rules for classifying sputtered species are given in the Method section, alongside a mathematical description of the Bohdasnky function and its corrections used throughout this work.

## Results

**Fig. 1:**
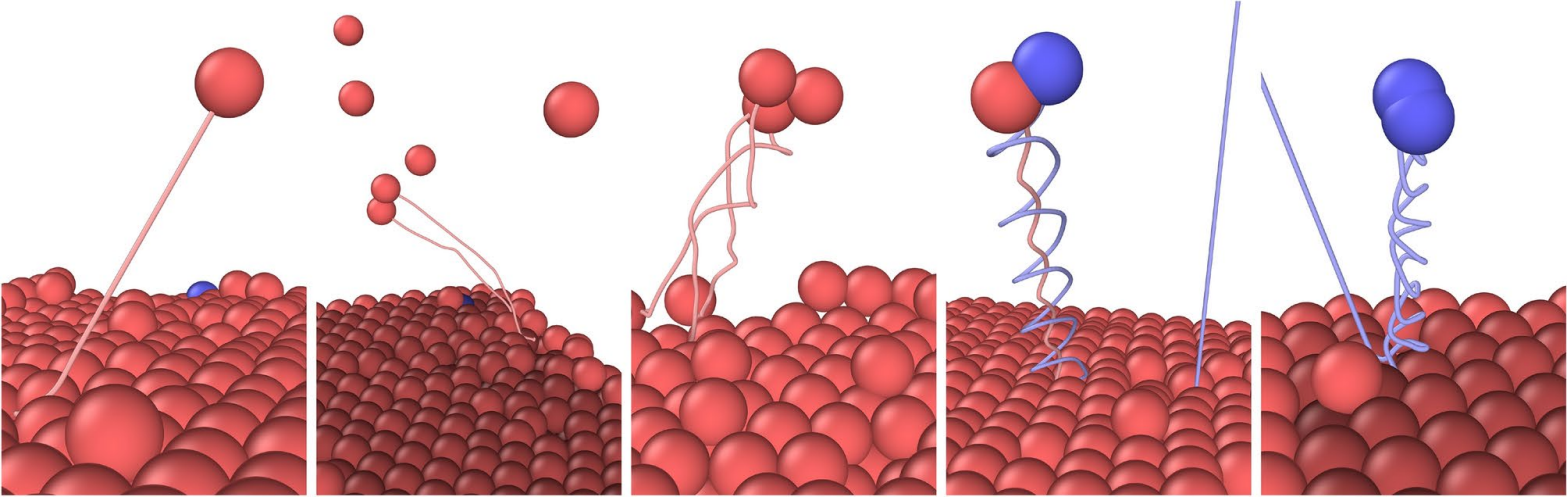
Sample trajectory for every type of sputtered species observed during this study (from left to right: atomic Be, Be$_2$ dimer, Be$_3$ trimer, BeD dimer, and a D$_2$ dimer). These sputtering events, rendered using OVITO^27^, were observed at various bombarding deuterium energies $T_\textrm{D}$ up to 500 eV and a fixed lattice temperature $T_{\textrm{Be}}$ = 300 K. Per-atom trajectories are colour-coded by atom type (Be in red, D in blue) and are shown only for the bombarding ion (if in the field of view) and the sputtered species of interest.

As part of the study, five simulated beryllium lattices with surface temperatures $T_{\textrm{Be}}$ ranging from 300 K to 1100 K underwent a total of 14,992 non-cumulative bombardments (each bombardment starts from the same state and does not influence successive bombardments) by deuterium atoms with energies corresponding to deuterium plasma ion temperatures $T_\textrm{D}$ ranging from 5 eV to 150 eV, with a particular focus in the 10-25 eV energy range near the expected beryllium sputtering threshold energy (9.8 eV to 26.2 eV reported^26^, or 14.1 eV from Eq. (5)). For investigating isotope effects, an additional 2605 tritium bombardments with the same energy range were carried out on a single $T_{\textrm{Be}}$ = 300 K lattice. Each lattice was equilibrated at its respective energy and prepared using cumulative bombardments by deuterium or tritium ions as described in the Methods section. Bombarding ion trajectories were classified as reflected, sorbed or transmitted to evaluate possible sputtering preference, and the sputtering events of atomic Be, the Be$_2$, BeD and BeT dimers, as well as the Be$_3$ trimer were recorded, as illustrated in Fig. 1. The sputtering yield of each of these species, as well as its dependence on the bombarding ion isotope, energy and trajectory, are presented in this section. Although sputtering of the D$_2$ and T$_2$ dimers has also been observed, its yield primarily depends on the quantity of trapped hydrogen in the beryllium lattice, and as such is only briefly discussed in this section.

Although it is known that the height of the lattice in this MD model limits the ion energy range that can be effectively investigated (as explored further in the Discussion section), an additional 6169 bombardments were performed in the “extended energy regime” with deuterium and tritium energies of up to 1 keV, focusing on the fraction of sputtered species by reflected or sorbed bombarding ions to study preferential sputtering behaviour over a larger range of energies. As part of model validation and to ensure that the reported results are not strongly influenced by the choice of MD simulation parameters, 7388 bombardments by deuterium were also performed on lattices prepared at cumulative bombardments of higher energy, with an increased interatomic potential cutoff distance, and with two different thermal damping times; each tested validation system did not show significant variation in fitted sputtering yield parameters, as discussed in more detail in the Methods section.

### Sputtering of atomic Be

**Fig. 2:**
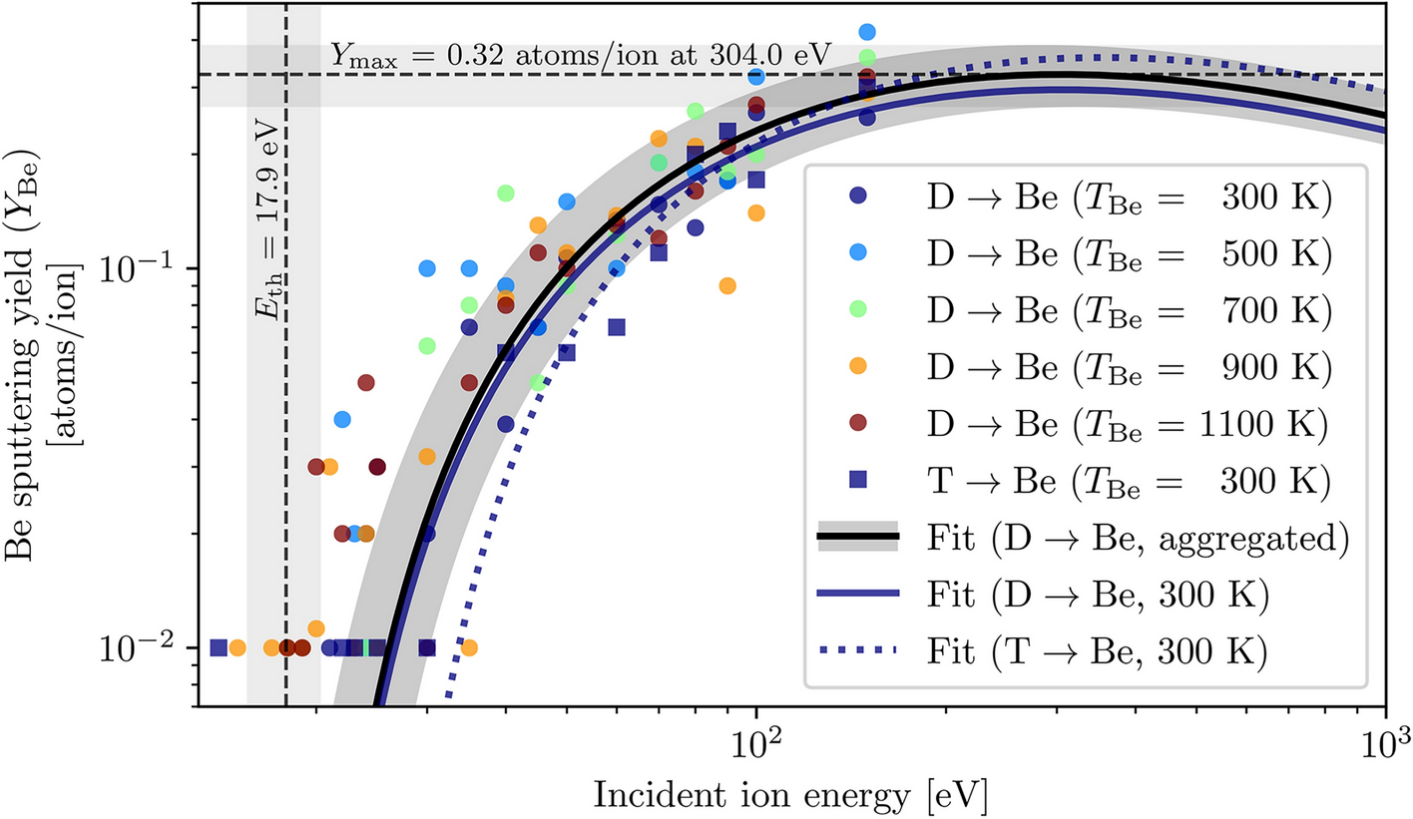
Sputtering yield of atomic beryllium by 5–150 eV deuterium and tritium ions from systems with beryllium surface temperatures between 300 K and 1100 K. A universal sputtering yield curve as defined in Eq. (8) has been fitted to aggregated sputtering data by deuterium across all available target temperatures (solid black line), as well as the beryllium sputtering yields by deuterium and tritium at a single surface temperature of 300 K (solid blue and dashed blue lines, respectively). The shaded regions represent three standard deviations of fitting error for the aggregated sputtering yield curve and the corresponding fitted/derived parameters. Sub-threshold sputtering events of atomic Be (e.g. where a sputtered BeD dimer disassociates into atomic Be and D, as discussed in the following subsection and illustrated in Supplementary Media 1) are rare but possible.

The simulated sputtering yield of atomic beryllium by deuterium and tritium ions as shown in Fig. 2, where each data point represents the ratio of sputtered atoms per number of bombarding ions of a specific energy, was fitted to the universal sputtering relation introduced in Eq. (2) (particularly its corrected form as specified in Eq. (8)); the surface approach angle of the bombarding ion was kept fixed at the optimised value of $\alpha$ = 15.94$^\circ$ during fitting as discussed in the data fitting subsection, and the fitted values of the energy-independent factor *Q* and the sputtering threshold energy $E_{\textrm{th}}$ are reported, alongside two useful derived parameters: the maximal sputtering yield $Y_{\textrm{max}}$ and the ion energy $E_{\textrm{max}}$ at which this maximum occurs.

For deuterium projectile ions, fitting was initially performed individually for each of the five available beryllium target temperatures between 300 K and 1100 K to investigate potential thermal effects on the sputtering yield. However, as no such effect was observed in either of the fitted parameters over the investigated range of target temperatures, the data was aggregated and fitted as a single “D$\rightarrow$Be” dataset to increase the available statistics and therefore reduce the fitted error of the reported parameters, as shown in Fig. 2 and summarised in Table 1. This lack of thermal effects on the sputtering yield is corroborated by available experimental data^25^, but may also be a limitation of the model, as discussed in the introductory model publication^22^.

As the number of bombardment events for each ion energy is finite and averages around 100 bombardments per data point in this dataset, this imposes a limit on the minimum measurable sputtering yield of around $Y_{\textrm{Be}} = 10^{-2}$ atoms/ion; this is visible in Fig. 2, and gives the impression that the universal sputtering function does not fit well to the data near the sputtering threshold energy. However, it should be noted that due to the high frequency of points around the threshold energy, a large fraction of points that are not visible on the log scale lie at $Y_{\textrm{Be}} = 0$ atoms/ion; combined with the fact that the influence of each point on the fitted sputtering yield curve is weighted according to the total number of bombardments used to compute it, the fitting routine can be considered to be reasonably robust.

Concerning tritium projectile ions and isotope effects on sputtering yield, MD simulations were performed for only a single beryllium target temperature of 300 K, the dataset for which was fitted in the same manner as the deuterium dataset; the fitted parameters are reported in Table 1, and show a notable increase in the sputtering threshold energy for T$\rightarrow$Be compared to D$\rightarrow$Be consistent with the theoretical prediction shown in Fig. 8, as well as a small increase in *Q* as reported in literature^26^.

**Table 1:** Fitted and derived beryllium sputtering yield parameters, as well as their corresponding one standard deviation errors, for deuterium and tritium projectiles impacting a beryllium target. Although partially redundant, the non-aggregated D$\rightarrow$Be dataset for a 300 K beryllium target is included for ease of comparison with the corresponding T$\rightarrow$Be dataset.

	*Q* (atoms/ion)	$E_{\textrm{th}}$ (eV)	$Y_{\textrm{max}}$ (atoms/ion)	$E_{\textrm{max}}$ (eV)
D$\rightarrow$Be (aggregated)	1.23 ± 0.06	17.9 ± 0.8	0.32 ± 0.02	304 ± 7
D$\rightarrow$Be ($T_{\textrm{Be}}$ = 300 K)	1.12 ± 0.09	18.1 ± 1.1	0.30 ± 0.03	305 ± 10
T$\rightarrow$Be ($T_{\textrm{Be}}$ = 300 K)	1.45 ± 0.13	23.6 ± 1.8	0.36 ± 0.04	353 ± 15

**Fig. 3:**
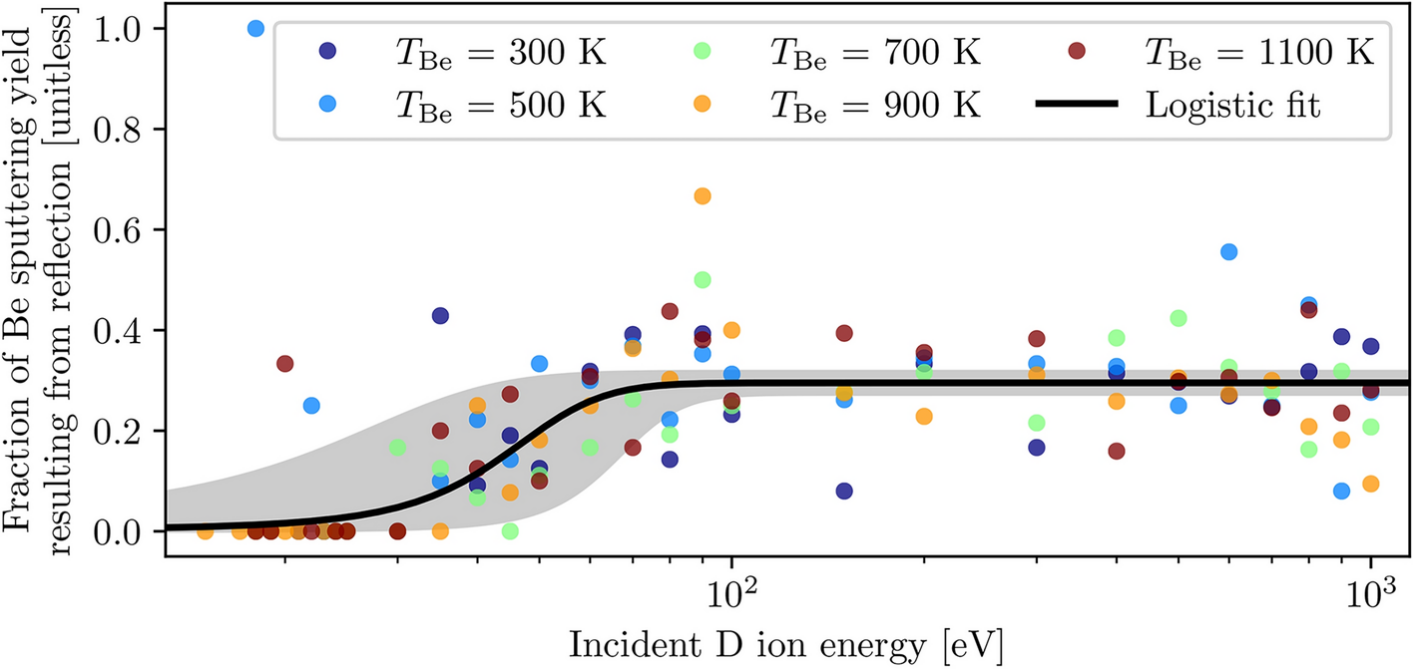
Preferential sputtering of atomic Be by D ions, where each point represents the fraction of sputtering yield resulting from reflection of the incident D ion at a single ion energy $T_\textrm{D}$ and target temperature $T_{\textrm{Be}}$ (for example, a value of 0.2 suggests that 20% of the sputtering events were the result of reflection of the bombarding ion, and the remaining 80% resulted from adsorption or absorption of the ion). The aggregated data was fitted to a logistic curve defined in Eq. (1) (*L* = 0.30±0.01, *k* = 0.1±0.1, $x_0$ = 44±7 eV), where the shaded region represents three standard deviations error of fit in *L* and $x_0$.

Each bombardment event results in either the reflection of the projectile ion from the target surface, or the sorption of the ion onto/into the target lattice; this ion trajectory classification is recorded for every sputtering event to deduce whether certain species preferentially sputter via a specific mechanism. Aggregated D$\rightarrow$Be sputtering data for atomic beryllium in the extended range indicates that this species strongly prefers sputtering via sorption of the bombarding ion, as illustrated in Fig. 3. At projectile ion energies below 44±7 eV, sputtering of atomic Be is almost entirely associated with sorption, while reflection accounts for approximately 30±1% of sputtering above this ion energy; over the investigated bombarding ion energy range, this preferential sputtering behaviour appears to follow a logistic curve of the form

1 $$\begin{aligned} f(x) = \frac{L}{1 + \exp {(-k(x - x_0))}} \end{aligned}$$

where *L* represents the curve’s maximum value (largest fraction of sputtering yield resulting from reflection of incident ions), *k* is the steepness of the curve and $x_0$ is the function’s midpoint. The authors make no claim that the logistic function represents a physical description of the underlying system; it is utilized solely as a mathematical tool to parametrize the observed trends and enable meaningful quantitative analysis. Additionally, there appear to be no isotope effects associated with preferential sputtering of atomic Be, as the T$\rightarrow$Be dataset appears to follow the same curve as fitted to the D$\rightarrow$Be dataset in Fig. 3.

### Sputtering of BeD and BeT dimers

Unlike atomic beryllium, the sputtering of the beryllium deuteride (BeD) and beryllium tritide (BeT) dimers does not follow the universal sputtering relation and instead sputters independently of the bombarding ion energy in the 5-1000 eV ion energy range. Sputtering of BeD has been observed at incident ion energies as low as 10 eV (sub-threshold when compared to sputtering of atomic Be) and as high as 900 eV with a sputtering yield of approximately 1 dimer for every 681 bombardments at $T_{\textrm{Be}}$ = 300 K (sample size of 5447, averaged over the entire extended ion energy range of 5 eV to 1 keV). Isotope effects also appear to be negligible, with the sputtering yield of BeT at the same target temperature of $T_{\textrm{Be}}$ = 300 K estimated to be 1 dimer for every 701 bombardments (sample size of 3505).

Notably, both dimers show strong preferential sputtering behaviour, with all recorded sputtering events associated exclusively with the reflection of the incident ion. Surface atoms are “pulled out” by the bombarding ion on reflection akin to the swift chemical sputtering (SCS) process^28^, and the sputtered dimers therefore exit the surface near the ion impact location, as was illustrated in Fig. 1, unlike atomic Be which is primarily ejected out of the lattice due to a collision cascade. As an indirect result of this, BeD and BeT dimer sputtering has been observed to cause minimal damage to the lattice since bombarding ions transfer less of their initial energy to the lattice on reflection as opposed to sorption. Similar behaviour has also been observed with the D$_2$ and T$_2$ dimers, both in terms of preferential sputtering on reflection and independence of yield on ion energy. The magnitude of the sputtering yield of D$_2$ and T$_2$, however, strongly depends on the initial concentration of D or T atoms near the surface, which in turn strongly depends on the conditions at which the lattice was prepared, and as such is not reported here.

There is also some indication of minor thermal effects on the sputtering yield of BeD, which has been observed to steadily increase with temperature, as summarised in Table 2. Surface atoms of systems with greater lattice temperatures are more energetic and therefore more likely to exceed the surface binding energy when imparted with excess energy from a bombarding ion. The T$\rightarrow$Be dataset does not contain data at other target temperatures, so no observations with regards to thermal effects on the yield of BeT can be made.

**Table 2:** Computed sputtering yields of the BeD and BeT dimers at all available target temperatures, averaged across data from bombarding ion temperatures of 5–1000 eV. A consistent increase in sputtering yield with target temperature can be noted, however, the outlier in $T_{\textrm{Be}}$ = 900 K may also be indicative of the high noise present throughout the data, and suggests the need for a larger sample size before conclusions can be drawn.

	D$\rightarrow$Be: BeD					T$\rightarrow$Be: BeT
	$T_{\textrm{Be}}$ = 300 K	$T_{\textrm{Be}}$ = 500 K	$T_{\textrm{Be}}$ = 700 K	$T_{\textrm{Be}}$ = 900 K	$T_{\textrm{Be}}$ = 1100 K	$T_{\textrm{Be}}$ = 300 K
Sputtering yield (dimers/ion)	$1.5 \times 10^{-3}$	$2.4 \times 10^{-3}$	$3.5 \times 10^{-3}$	$1.6 \times 10^{-3}$	$4.0 \times 10^{-3}$	$1.4 \times 10^{-3}$
Sample size (bombardments)	5447	3689	3673	3752	3700	3505

### Sputtering of the Be$_2$ dimer and Be$_3$ trimer

**Fig. 4:**
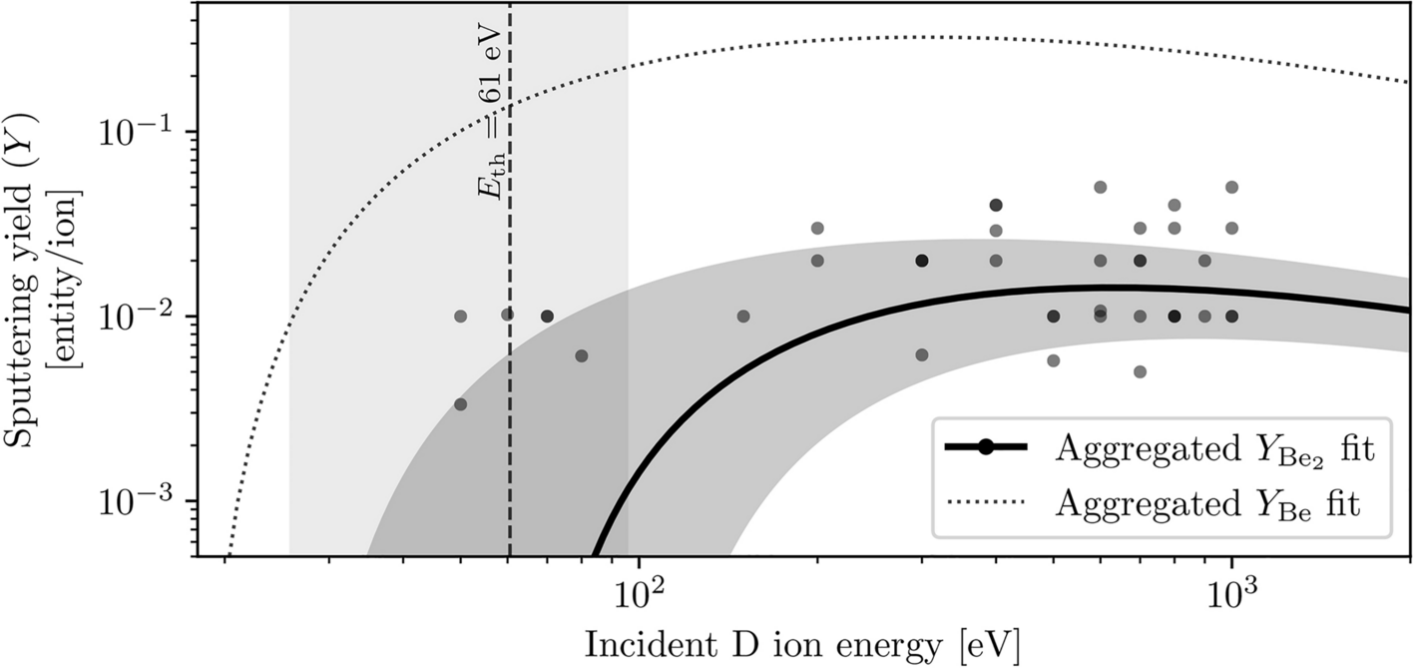
Sputtering yield of the Be$_2$ dimer by D ions, aggregated across all target temperatures and fitted to the universal sputtering relation (solid line), with the shaded area representing three standard deviations error of fit. Although the data does not appear to fit well to the curve, the fitting routine takes into account the density of zero-value sputtering yield data points which are not shown. A fitted sputtering yield curve for atomic Be (dashed line) is included for ease of comparison of sputtering yield magnitudes.

As with atomic Be, the sputtering yield of the Be$_2$ dimer is dependent on the incident ion energy, and while the universal sputtering yield relation is not directly applicable for computing the sputtering yield of dimers, it approximates the energy dependence of Be$_2$ sputtering yield well, as shown in Fig. 4. The dimer was first observed at deuterium ion energies of 50 eV, and to provide some comparison against atomic Be, fitting the extended energy D$\rightarrow$Be dataset to Eq. (8) reports a sputtering threshold energy $E_{\textrm{th}}$ = 61±12 eV, *Q* = 0.08±0.01 dimers/ion, with a maximum sputtering yield of 0.014±0.003 dimers/ion at $T_{\textrm{D}}$ = 630±80 eV. The large errors are due to the relatively low sputtering yield of Be$_2$ and therefore limited available statistics; additionally, as a result of the increased $E_{\textrm{th}}$ and decreased *Q*, the extended energy range is likely insufficient for estimating the maximum sputtering yield of Be$_2$ without some extrapolation. In line with previously observed isotope effects on the sputtering threshold energy, the first sputtered Be$_2$ dimer in the T$\rightarrow$Be dataset was recorded at an increased ion energy $T_{\textrm{T}}$ = 90 eV. Notably, the Be$_2$ dimer shows no preferential sputtering behaviour in the extended ion energy range, being sputtered equally by both reflected and sorbed ions.

The Be$_3$ trimer was first observed to sputter by D ions at $T_{\textrm{D}}$ = 400 eV, and has an estimated sputtering yield of $2.5 \times 10^{-4}$ trimers/ion in the bombarding D ion energy range of 400-1000 eV (averaged across all target temperatures in the D$\rightarrow$Be dataset, with a total sample size of 20,261). Sputtering of the trimer is sufficiently infrequent that neither preferential sputtering nor isotope effects can be estimated, even in the extended energy range.

## Discussion

**Fig. 5:**
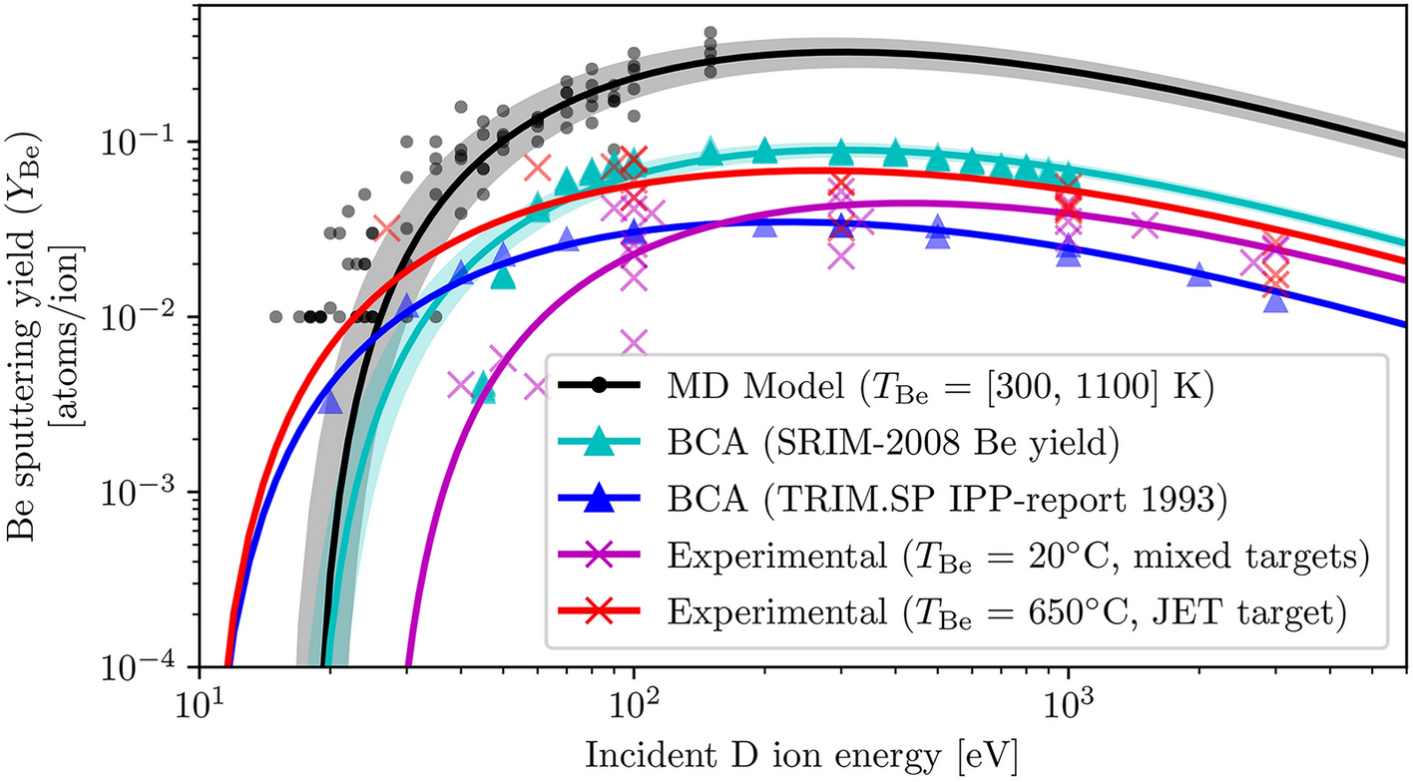
Comparison of the sputtering yield of atomic Be predicted by the MD model to data from other sources. All external data plotted here has been collected in and sourced from an IPP 9/82 sputtering data document^26^. The sputtering yield of atomic Be by D ions in the energy range (5–150 eV) was aggregated across all available target temperatures and fitted to the corrected universal sputtering yield given in Eq. (8) (black). BCA data has been computed using the SRIM-2008 software, where the reported Be yield at ion energies of 5–1000 eV was aggregated across all surface approach angles up to 25$^\circ$ and fitted to corrected universal sputtering relation (cyan). External BCA data was computed using the TRIM.SP package and the corresponding pre-fitted sputtering yield parameters were substituted into the standard sputtering relation without angular corrections (blue). For external experimental data, measured during the bombardment of mixed beryllium targets at room temperature (magenta) and beryllium JET targets at $650^\circ$C (red), pre-fitted parameters were provided which were also plotted using the standard sputtering relation (see Eq. 2). All shaded regions represent three standard deviations of fitting error, where fitting was performed.

To evaluate the effectiveness of the MD model and the reported sputtering behaviours, the predicted sputtering yield of atomic Be is compared against similar data from alternative sources. This metric was chosen as molecular sputtering data is not prevalent across literature and cannot be computed easily using BCA methods via SRIM; similarly, the preferential sputtering statistics reported here are novel to the best of the authors’ knowledge. Figure 5 compares the sputtering yield data of atomic Be by D ions from five sources: MD data reported in this work (black), BCA Be yield computed using SRIM-2008 (cyan), previously reported BCA data computed using TRIM.SP^26^ (blue), experimental data collected from mixed beryllium targets at room temperature^26^ (magenta) and experimental data collected from a JET target at $650^\circ$C^26^ (red).

The BCA data computed as part of this work modelled a beryllium slab with a thickness of 1$\upmu$m and a smooth surface, bombarded by 99,999 deuterium atoms (hydrogen with a mass of 2.014 amu) with energies of 5-1000 eV at 6 surface approach angles between 0$^\circ$ and 25$^\circ$ (inclusive), for a total of 9 million bombardments using the “surface sputtering” calculation type of SRIM-2008. Although this represents a statistically significant dataset with a distribution of bombardment angles similar to that simulated by the MD model, it is important to note that more advanced BCA codes such as SDTrimSP-3D^29^ or SPRAY^30^ which also consider the target surface morphology may provide an even better comparison to the rough surface modelled using MD^31^.

A general variation of parameters can be noted, with the estimated sputtering threshold energy $E_{\textrm{th}}$ within the range of experimentally reported and computationally derived values (and reasonably close to the theoretically predicted value in Fig. 8); the energy-independent factor *Q*, and as a result the maximum sputtering yield $E_{\textrm{max}}$, appears to be overestimated by the MD model by up to an order of magnitude compared to experimental data. The overestimation is likely caused by the interatomic potential used as part of the MD model; however, validation datasets generated using a modified potential with an increased cutoff distance and a range of thermal damping times showed no significant difference in the fitted *Q*, as discussed further in the Method section. More importantly, no other interatomic potential for the interaction of Be-H was available that would be more accurate as well as computationally inexpensive enough to generate datasets with similar statistical significance as those reported here. Additionally, the isotope effects of increased $E_{\textrm{th}}$ with increased isotope mass predicted by the model are in line with the expected theoretical behaviour (see Eqs. 5 and 9), and the predicted effect of a small increase in *Q* is in line with experimentally observed behaviour^26^.

It has been noted that the accuracy of the sputtering yield predicted by this model degrades at bombarding ion energies above 150 eV. Primarily, this is a limitation imposed by the size of the model, with higher energy ions permeating through the lattice and desorbing via the lower surface before being able to deposit sufficient energy into the lattice. It is known that bombarding hydrogen ions tend to reflect from internal lattice layers and travel back towards the surface after dissipating sufficient energy^22^—a process that can no longer take place if total ion permeation occurs, suggesting an underestimation of the sputtering yield for species that depend on such a collision cascade for sputtering.

Additionally, at ion energies close to 1 keV, bombarding ions reflecting from the lattice surface exit the simulation box faster than the resulting collision cascade can propagate, with the simulation terminating sooner than sputtered atoms can move sufficiently away from the influence of the surface to be considered sputtered. However, at such ion energies, an additional overestimation of sputtering yield also arises due to the deformation of the surface on ion impact; if the surface deforms sufficiently, atoms at a pre-computed distance above the surface will be considered as sputtered despite still being under the influence of the surface (in an extreme scenario, a chain of atoms that is still directly connected to the lattice, but the top of which is above the threshold distance for sputtering). To overcome this, more advanced counting rules for sputtered atoms would be needed, computing not only if the sputtered atom is within the influence of the surface, but whether the atom is within the interatomic potential cutoff distance of any neighbouring atoms which would themselves have to clear the threshold distance away from the surface.

The concept of outgassing is also important from the perspective of minimising the trapped tritium inventory in beryllium PFCs. Although the timescales of the MD study conducted here do not allow for the study of outgassing directly, it should be noted that sputtering of lone D and T atoms has also been observed across a wide range of bombarding ion energies. This hints at the presence of a sub-threshold ion-assisted outgassing process that does not introduce molecular impurities into the plasma, which may warrant further study.

## Concluding remarks

In this study, the sputtering processes of beryllium species by hydrogenic plasma were statistically investigated under conditions resembling fusion PFCs exposed to burning plasma, with a particular focus on determining preferential sputtering behaviour and observing thermal and isotope effects on sputtering yield. Notably, the estimated yields and required conditions for sputtering of beryllium dimers and trimers are reported for the first time, as well as novel insight into the sputtering processes of certain species due to their strong preferential sputtering behaviour: hydrogenous dimers such as BeD and D$_2$ sputter exclusively on bombarding ion reflection and do not rely on momentum transfer to the lattice - as a result, they experience little to no dependence on ion energy or isotope over the investigated energy range; non-hydrogenous species such as Be and Be$_2$ that do not show sputtering preference or exhibit partial preference towards sorption of the bombarding ion instead rely on collision cascades for sputtering - such species sputter according to the universal sputtering relation and exhibit isotope effects associated with variations in momentum of the projectile ion. Proposed avenues of future work involve a thorough analysis of collision cascades in the existing MD data to quantify non-sputtering lattice damage and possible thermal annealing effects.

## Methods

The data presented and analysed throughout this report was generated using an existing MD model initially developed for the study of ion penetration depth profiles in beryllium^22^. In this section, we provide only a brief discussion of the model and its parameters used during the equilibration, surface preparation, and production stages. Instead, our focus shifts towards the interatomic potential, validation procedure, and the counting rules for sputtered species.

Where applicable, sputtering yield data was fitted to Yamamura’s angular correction^32^ of the modified stopping power^33^ Bohdansky universal sputtering relation^23^, as discussed in latter parts of this section. At a constant ion bombardment angle, the sputtering relation is a function of two commonly reported^24^ parameters: the energy-independent scaling factor *Q* and the sputtering threshold energy $E_{\textrm{th}}$, the significance of which is also discussed in this section. Additional functions are also given for estimating the expected sputtering threshold energy $E_{\textrm{th}}$ from first principles.

### Molecular dynamics model

The MD model used throughout this study simulates 9786 beryllium atoms initially arranged in a hexagonal close-packed (HCP) lattice structure, exposing a 40 Å by 40 Å periodic surface with a non-periodic depth of 50 Å, oriented such that the “ABAB” planes are stacked along the non-periodic axis and the (001) HCP plane is parallel to the surface. Interactions between all beryllium and hydrogen atoms are governed by an existing analytical bond-order potential (ABOP)^34^ modified to suit the 3-body Tersoff formalism^35^ implemented by the LAMMPS Molecular Dynamics Simulator^36^. The Tersoff parameters that can be used to recreate this interatomic potential are given in Table 3, where deuterium and tritium isotopes are simply treated as mass variants of the hydrogen atom.

**Table 3:** Tersoff parameters^35^ of the interatomic potential used throughout this study. The potential was initially introduced with the ABOP formalism for describing the interactions of atoms in a Be–C–H system^34^, and was rewritten here to suit the Tersoff formalism^35^ utilised by LAMMPS^36^.

	*m*	$\gamma$	$\lambda _3$	*c*	*d*	$\cos {(\theta _0)}$	*n*	$\beta$	$\lambda _2$	*B*
Be–Be	1.0	$4.78701 \times 10^{-7}$	1.7	32.32797	0.05265	$-0.82658$	1.0	1.0	1.026191	13.915215
Be–H	1.0	$0.14\ \ \,$	0.0	0.0057	0.002	$-1.0$	1.0	1.0	1.877942	48.118327
H–H	1.0	12.33	0.0	$0.0\ \,$	1.0	$-1.0$	1.0	1.0	1.795631	31.379342

	*R*	*D*	$\lambda _1$	*A*						
Be–Be	2.685	0.223	3.193168	367.830049						
Be–H	1.75	0.15	5.633826	2441.637010						
H–H	1.40	0.30	4.207524	80.070348						

All modelled atoms were initialised with random velocities sampled from a uniformly distributed ensemble representing the target lattice temperature $T_{\textrm{Be}}$, and the system was advanced until an equilibrium state was reached (minimum 200 picoseconds simulation time, change in average total energy per beryllium atom no more than 1meV in the last 100 picoseconds^22^). Time integration was performed using the Verlet algorithm, with a Nosé–Hoover thermostat sampling from the canonical NVT ensemble during the equilibration period. Dynamic time-stepping (LAMMPS dt/reset every 10 timesteps, maximum atom displacement of 10 femtometers per timestep) between 1 attosecond and 1 femtosecond was used to reduce the overall computational cost of the model while ensuring system stability and accuracy of high-velocity interatomic interactions (such as those during initial equilibration timesteps, as well as during subsequent lattice bombardments). A total of five different systems were equilibrated at lattice temperatures $T_{\textrm{Be}}=$ 300 K, 500 K, 700 K, 900 K and 1100 K in order to investigate potential surface temperature effects on sputtering behaviour.

Each equilibrated system, which initially featured an ideal relaxed beryllium surface, underwent surface preparation emulating the exposure of the surface to hydrogenic plasma. During this process, the surface was cumulatively bombarded by 50 eV deuterium and tritium ions, inducing damage to the surface and upper bulk of the lattice in the form of lattice dislocations and vacancies, adsorbed interstitial hydrogen atoms, and sputtered/redeposited beryllium species. Each projectile ion was created at a random position 25 Å away from the surface with its velocity vector oriented to a random but constrained surface approach angle^22^, which was assumed to be uncharged at this stage as a result of the Auger neutralisation process^22,37^.

All projectile ions sample from the NVE ensemble and impart their energy onto the beryllium surface on impact. As the top section of the lattice also operates under the NVE regime, this energy is gradually distributed throughout the bulk of the lattice towards the lower layers. The bottom 5 Å of the lattice, acting as a heat sink that dissipates this excess energy, operates under the NVT regime and is thermostatted to the initial equilibrium temperature of the lattice, such that the system eventually returns to an equilibrium state at the target lattice temperature. The thermal relaxation parameter of the thermostat, which controls the rate at which the energy is dissipated and ultimately affects the time it takes for the system to re-equilibrate, has been left at the default value of 100 timesteps, although alternative values have been tested as per the validation process. To save computational time and increase the maximum feasible number of cumulative bombardments that can be performed, successive bombardments are triggered as soon as the system is considered re-equilibrated; it should be noted that the effective preparatory ion flux is therefore significantly higher than would be expected in typical fusion reactor conditions. As momentum is imparted onto the lattice by each bombardment, to prevent the lattice from drifting in the simulation box, the average system momentum is periodically rescaled in the non-periodic *z*-axis, followed by a re-centring of the lattice to its initial centre-of-mass^22^; rather than fixing the lower lattice monolayers in space, as is common across similar molecular dynamics studies^38,39^, the momentum rescaling option was chosen to maximise the available penetration depth, enable constant use of the heat sink, and to avoid overestimation of sputtering yields caused by enforcing the reflection of bombarding ions from the fixed lattice monolayer. Each system is cumulatively bombarded 20 times, with a 25 picosecond wait between successive bombardments.

A total of 6 systems were prepared in this manner: 5 by deuterium ions at a range of lattice temperatures, and 1 by tritium ions at room temperature ($T_{\textrm{Be}}=$ 300 K). The state of each system is saved, and all non-cumulative production bombardments start from these states. Non-cumulative bombardments are performed similarly to cumulative ones: a projectile ion is randomly created 25 Å above the surface, with an energy equivalent to the plasma ion temperature being studied and a velocity vector rescaled such that the ion impacts the lattice at a random angle constrained to a range of valid surface approach angles. The simulation is ended after a fixed period of 1 million timesteps or if any atom leaves the simulation box (the latter assumes a sputtering event, but a reflected bombarding ion can also trigger this condition); at the penultimate simulation time-step, all atoms (except the bombarding ion) with a *z*-coordinate above the field of influence of the surface are considered sputtered. The threshold *z*-coordinate is computed as the interatomic potential cutoff distance ($R + D$ = 2.908 Å for a Be-Be interaction) above the pre-computed position of the surface after preparation; as described previously^22^, the average height of the surface has to be known to a reasonable degree of accuracy to ensure that all atoms counted as sputtered would not be able to return to the surface if the simulation was allowed to run for longer.

If a sputtered atom is within the cutoff distance of 2.908 Å away from one or more other atoms, the entity is counted as a single sputtered dimer or trimer. As such, the sputtering yield for a total of 6 different species (10 if including their isotopologues) is reported for each bombardment: atomic Be, atomic D and T, the BeD and BeT dimers, the Be$_2$ dimer, the D$_2$ and T$_2$ dimers, as well as the Be$_3$ trimer. While it is possible for dimers and trimers to form above the surface from individually sputtered atoms, due to the variation of the sputtered atoms’ momentum and angle and absence of any long-distance attractive force, such interactions are extremely unlikely, and thus virtually all recorded dimers and trimers were sputtered directly and remained stable for the duration of the simulation (see Fig. 1). Additionally, the trajectory of each bombarding ion can be classified into one of three categories: reflection (bombarding ion reflects off the top lattice surface and exits the simulation box), sorption (bombarding ion remains absorbed on the top lattice surface or is absorbed into the bulk of the lattice), and transmission (bombarding ion travels through the bulk of the lattice, desorbs via the lower lattice surface, and exits the simulation box). The latter is a limitation due to the height of the lattice in the non-periodic *z*-dimension and places an upper limit to the bombarding ion energy range that can be investigated before ion transmission becomes dominant. However, for the sake of studying preferential sputtering, transmitted ions are counted as sorbed (or more strictly, they are counted as *not* reflected).

To validate the behaviour of the model and ensure that the reported sputtering behaviour is not strongly influenced by internal MD model parameters or conditions at which the systems were prepared, several additional validation systems were prepared and used for production bombardments in tandem with the aforementioned 6 final systems. Validation systems such as one with an increased interatomic potential cutoff distance (*R* = 3.5 Å, $R + D$ = 3.723 Å), a system prepared by cumulative bombardments by D ions with a temperature of 200 eV ($4\times$ the nominal value of 50 eV used in production), and two systems prepared with thermal damping times of 10*dt* and 1000*dt* ($0.1\times$ and $10\times$ the nominal value of 100*dt*) were evaluated, and no significant changes in sputtering yield or preferential sputtering behaviour was noted. Various other MD system parameters were also optimised as part of the validation process: the system size in the periodic *x*- and *y*-dimensions was chosen to minimise finite-size effects, the system size in the non-periodic *z*-dimension was optimised to balance the maximum ion energy that can be accurately investigated against the computational cost necessary for the desired statistical sample size, and the minimum system preparation re-equilibration time was evaluated at a range of ion and lattice temperatures to minimise the simulation time and therefore maximise the feasible number of cumulative bombardments performed.

### Universal sputtering yield

A universal function describing the sputtering yield of monoatomic solids was proposed by Bohdansky in 1984^23,25^ as an extension of Sigmund’s theory of backward sputtering^40^. In the proposed function, the sputtering yield *Y* [atoms/ion] for any bombarding atom with energy $E_0$ [eV] (at normal incidence $\alpha = 0^{\circ }$, and neglecting sub-threshold sputtering such that $E_0 \ge E_{\textrm{th}}$) is defined as

2 $$\begin{aligned} Y(E_0,\ \alpha =0^{\circ }) = Q s_n(\epsilon ) \left( 1 - \left( \frac{E_{\textrm{th}}}{E_0} \right) ^ {2/3} \right) \left( 1 - \frac{E_{\textrm{th}}}{E_0} \right) ^ 2 , \end{aligned}$$

where *Q* (energy-independent factor [atoms/ion]) and $E_{\textrm{th}}$ (sputtering threshold energy [eV]) are fitting parameters used throughout this work, $s_n(\epsilon )$ is the nuclear stopping cross-section (a function of reduced energy $\epsilon$), and the latter terms represent corrections for bombarding energies near the threshold energy ($E_0 \approx E_{\textrm{th}}$). The reduced energy $\epsilon$ is defined as

3 $$\begin{aligned} \epsilon = \frac{E_0}{E_{\textrm{TF}}},\qquad E_{\textrm{TF}} = \frac{Z_1 Z_2 e^2}{a_\textrm{L}} \frac{M_1 + M_2}{M_2} , \end{aligned}$$

where $E_{\textrm{TF}}$ (eV) is the Thomas-Fermi energy, $Z_1$ and $Z_2$ are the projectile and target nuclear charges, $M_1$ and $M_2$ are the projectile and target atomic masses, $e = \sqrt{1.4400\ \text {eV}\cdot }$ Å is the elementary charge, and the interaction potential (Lindhard) screening length $a_\textrm{L}$ (Å) is given by

4 $$\begin{aligned} a_\textrm{L} = \left( \frac{9 \pi ^2}{128} \right) ^{1/3} a_\textrm{B} \left( Z_1 ^{2/3} + Z_2^{2/3} \right) ^{-1/2}, \end{aligned}$$

where $a_\textrm{B} = 0.5292$ Å is the Bohr radius.

The primary mechanism behind physical sputtering is strongly dependent on the mass ratio of the projectile and target atoms ${M_1/M_2}$, particularly at low bombardment energies $E_0$. Heavy ion sputtering (${M_1 \gg M_2}$) is the result of collision cascades generated directly by the projectile atom impacting the target, while light-ion sputtering (${M_1 \ll M_2}$) is largely the result of collision cascades caused by incident ions backscattering from within the bulk of the target^41^.

The sputtering threshold energy term $E_{\textrm{th}}$ of the universal sputtering relation represents a physical quantity that is particularly useful in nuclear fusion engineering; an energy below which no physical sputtering can occur (however, sub-threshold sputtering events are still possible due to chemical sputtering processes^28^). Empirical formulae for estimating the sputtering threshold energy at normal incidence ($\alpha =0^{\circ }$) have been proposed for both light-ion and heavy-ion sputtering^42^, where it is generally accepted across literature that ${M_1/M_2} < 0.2$ constitutes light-ion sputtering and ${M_1/M_2} > 0.3$ corresponds to heavy-ion sputtering^26,42^. These formulae, also visualised in Fig. 8, can be summarised as follows:

5 $$\begin{aligned} E_{\textrm{th}} (\alpha =0^{\circ }) = \left\{ \begin{array}{ll} \dfrac{E_\textrm{S}}{\gamma (1 - \gamma )} & \dfrac{M_1}{M_2} \le 0.3 \\ 8E_\textrm{S} \left( \dfrac{M_1}{M_2} \right) ^ {2/5} & \dfrac{M_1}{M_2} > 0.3 \end{array} \right. , \end{aligned}$$

where the original mass-ratio regime threshold of ${M_1/M_2 = 0.3}$ has been used, $E_\textrm{S}$ represents the surface binding energy, and $\gamma$ is the maximum energy transfer factor given by

6 $$\begin{aligned} \gamma = \frac{4 M_1 M_2}{(M_1 + M_2)^2}. \end{aligned}$$

It should be noted that although a solid material’s heat of sublimation is often used in literature as an approximation of its surface binding energy $E_\textrm{S}$^26,43^, the actual value of $E_\textrm{S}$ depends on factors such as surface orientation^44^ and roughness^45^, and may differ from its approximation by up to several electronvolts.

### Nuclear stopping cross-section and angular corrections

**Fig. 6:**
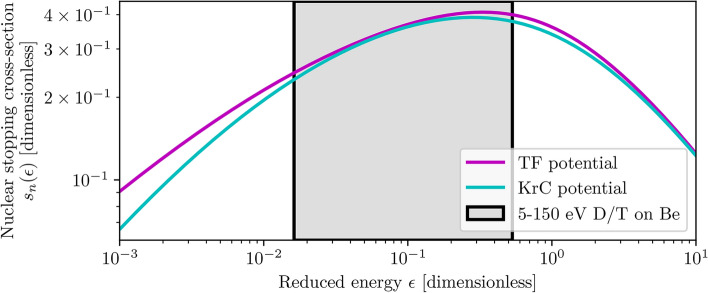
Comparison between nuclear stopping powers based on the Thomas–Fermi (TF) and Krypton–Carbon (KrC) potentials. The stopping powers diverge at reduced energies $\epsilon$ above $10^2$ (not graphed) and below $10^{-1}$. The reduced energy range of interest for 5–150 eV deuterium and tritium atoms incident on beryllium is highlighted in grey.

In the original definition of the universal sputtering relation^23^, an approximation of the Thomas-Fermi nuclear stopping power $s_n^{\textrm{TF}}$ has been used for the stopping cross-section term $s_n(\epsilon )$. It was later noted that a stopping power based on the Krypton-Carbon (KrC) potential $s_n^{\textrm{KrC}}$ is a better approximation at reduced energies $\epsilon < 100$^33^. The analytical expressions for both stopping powers are given in Eq. (7), and a comparison between them is graphed in Fig. 6. The difference between the two stopping powers in the reduced energy range of interest (ROI) for this work is small, however, $s_n^{\textrm{KrC}}$ is used as it does offer some improvement in the higher and lower parts of the ROI.

7 $$\begin{aligned} \qquad \quad s_n^{\textrm{TF}} = \frac{3.441 \sqrt{\epsilon } \ln {(\epsilon + 2.718)}}{1 + 6.355 \sqrt{\epsilon } + \epsilon (6.882 \sqrt{\epsilon } - 1.708)}\qquad s_n^{\textrm{KrC}} = \frac{0.5 \ln {(1 + 1.2288\epsilon )}}{\epsilon + 0.1728 \sqrt{\epsilon } + 0.008\epsilon ^{0.1504}} \end{aligned}$$

**Fig. 7:**
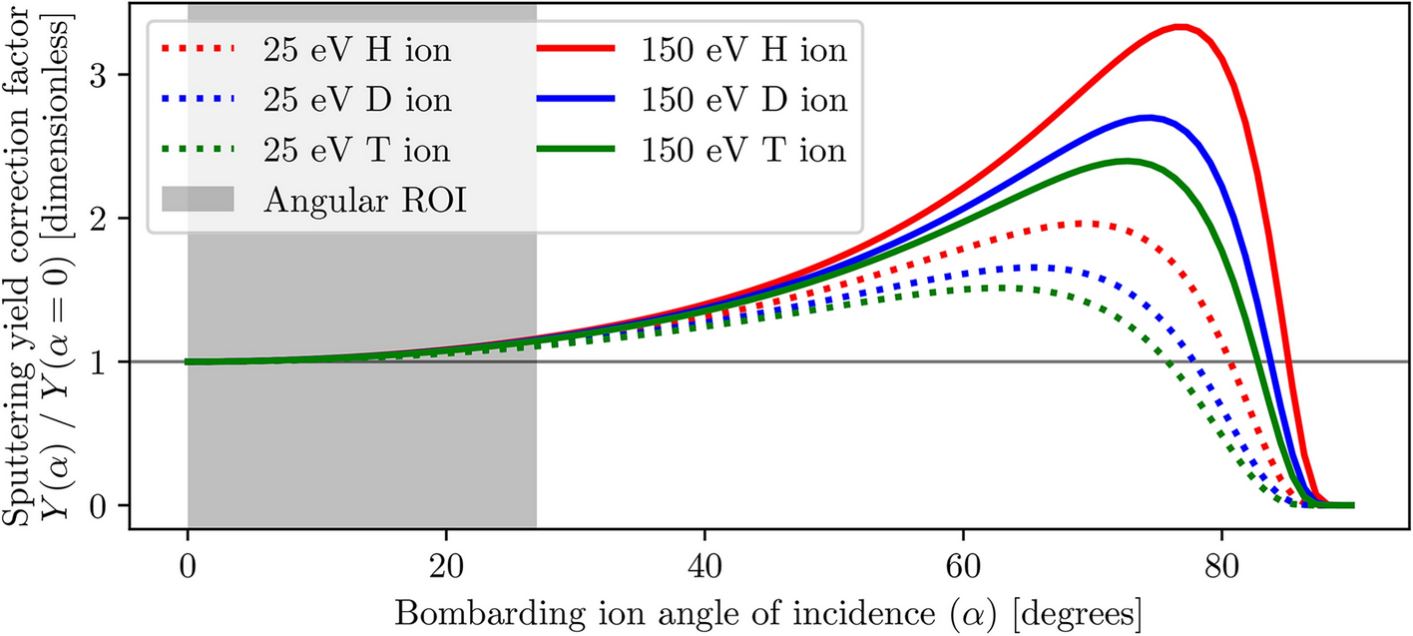
Magnitude of the sputtering yield correction factor $Y(\alpha ) / Y(\alpha = 0^{\circ })$ for non-normal ion incidence angles (see Eq. 8) at varying ion angles $\alpha$, energies $E_0$, and projectile masses $M_1$, and fixed target mass $M_2$ corresponding to beryllium. The correction factor increases with increasing $\alpha$ up to a maximum value of $\alpha _{\textrm{opt}}$ at which sputtering yield is the greatest. More energetic ions require a greater correction factor, as do lighter ion isotopes. For the ion isotopes and their incident angle ($\alpha \le 27^{\circ }$^22,46^, see grey region) and energy ($E_0 \le 150$ eV) ranges of interest explored in this study, the correction factor is small but not negligible.

In practice, plasma-surface interactions occur at a range of ion incidence angles^46^, therefore additional considerations also need to be made for sputtering at non-normal angles of incidence ($\alpha > 0^\circ$). An empirical formula that introduces an angular correction factor to the universal sputtering relation defined in Eq. (2) was proposed by Yamamura et al. in 1983^32^:

8 $$\begin{aligned} \begin{array}{rl} Y(E_0,\ \alpha ) & = Y(E_0,\ \alpha =0^{\circ }) (\cos {\alpha })^{-f} \exp {\left( f \left[ 1-(\cos {\alpha })^{-1} \right] \right) \cos {\alpha _{\textrm{opt}}}} \\ f & = \sqrt{E_\textrm{S}} [0.94 - {1.33 \times 10^{-3}} (M_2 / M_1)] \\ \alpha _{\textrm{opt}} & = \pi /2 - a_\textrm{L} n^{1/3} \left( 2 \epsilon \sqrt{E_\textrm{S} / \gamma E_0} \right) \end{array}, \end{aligned}$$

where the parameter *f* is introduced for clarity of the expression, $\alpha _{\textrm{opt}}$ represents the angle at which maximal sputtering occurs, and *n* (atoms/Å$^{3}$) is the density of the target. By inspecting Eq. (8), it can be seen that the magnitude of the correction factor depends not only on the angle of incidence $\alpha$ and projectile energy $E_0$, but also on the atomic masses $M_1$, $M_2$ and nuclear charges $Z_1$, $Z_2$ of the projectile and target material, respectively, as visualised in Fig. 7.

**Fig. 8:**
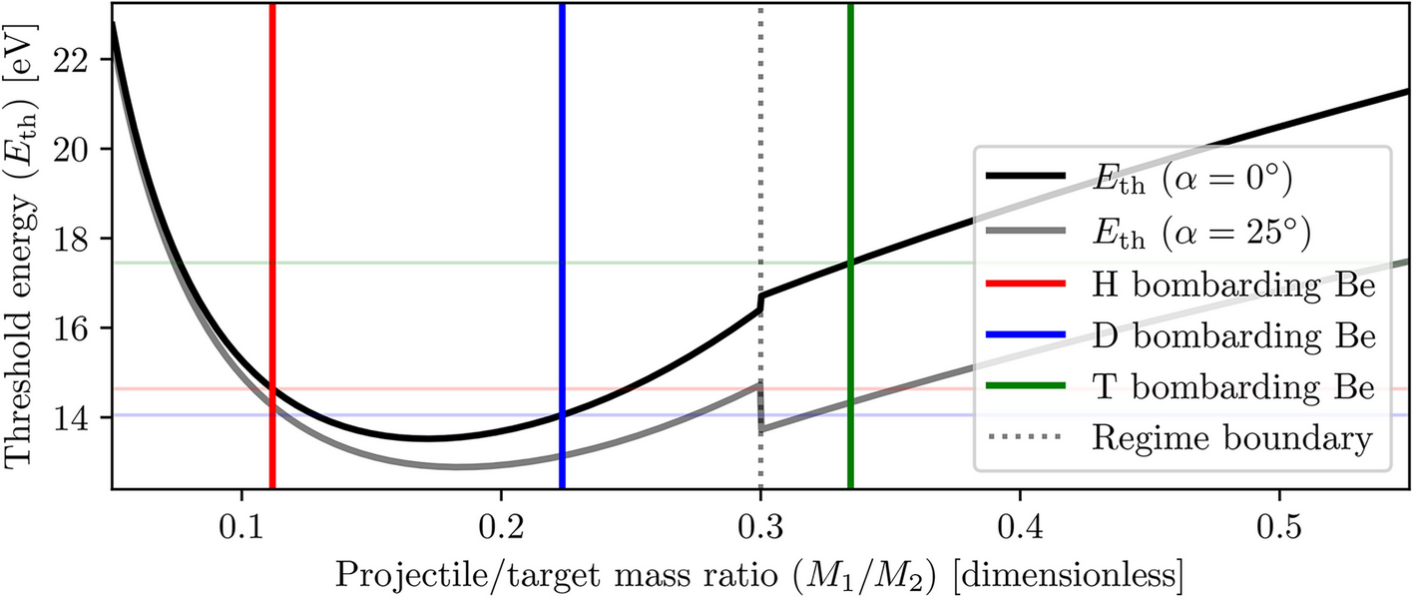
Estimated sputtering threshold energies $E_{\textrm{th}}$ at a range of projectile/target mass ratios $M_1/M_2$, for normal ($\alpha =0^{\circ }$, Eq. (5), black line) and non-normal ($\alpha =25^{\circ }$, Eq. (9), grey line) ion incidence angles. The discontinuity at $M_1/M_2 = 0.3$ represents the boundary between the light ion sputtering ($<0.3$) and heavy ion sputtering ($>0.3$) regimes. The mass ratios and the corresponding expected sputtering threshold energies at normal ion incidence of the projectile-target pairs of interest are shown in red (H$\rightarrow$ Be), blue (D$\rightarrow$ Be) and green (T$\rightarrow$ Be). Although presented as indicative only, it can be seen that within our parameter ranges of interest, the sputtering threshold energies decrease with increasing bombardment angles, especially at larger mass ratios.

Notably, modifications can also be made to Eq. (5) to allow for the estimation of sputtering threshold energies $E_{\textrm{th}}$ at non-normal but sufficiently low ($\alpha \le 70^{\circ }$)^32^ ion incidence angles^47^, as shown in Eq. (9) and Fig. 8. From this modified relation and within our ion incidence angle and mass ratio ROIs, it can be seen that increased incidence angles $\alpha$ are expected to marginally decrease threshold energies $E_{\textrm{th}}$ - a decrease which becomes more significant as the projectile/target mass ratio $M_{1}/M_{2}$ increases.

9 $$\begin{aligned} E_{\textrm{th}} (\alpha \le 70^{\circ }) = \left\{ \begin{array}{ll} \dfrac{E_\textrm{S}}{\gamma \left( 1 - \gamma \cos ^2 (\alpha / 2) \right) } & \dfrac{M_1}{M_2} \le 0.3 \\ 8E_\textrm{S} \left( \dfrac{M_1}{M_2} \right) ^ {2/5} \cos ^2 (\alpha ) & \dfrac{M_1}{M_2} > 0.3 \end{array} \right. \end{aligned}$$

### Fitting of sputtering yield data

The MD model simulates the bombardment of a beryllium target by projectile ions that impact the surface at a range of angles $\alpha$ uniformly distributed between $\alpha _{\textrm{min}} = 0^{\circ }$ and $\alpha _{\textrm{max}} = \tan ^{-1} \left( \sqrt{2} \tan (20^{\circ })\right) = 27.24^{\circ }$^22,46^, such that $\alpha \sim U \, \left[ 0^{\circ }, \, 27.24^{\circ }\right]$. As each reported sputtering yield data point represents an aggregation of sputtering events from several fixed-energy ion bombardments, the resulting sputtering yield curve corresponds to the average yield over the specified range of bombardment angles, rather than the single value of $\alpha$ compatible with Eq. (8). However, such value $\alpha _{\textrm{ave}}$ which *approximates* the average sputtering yield $Y_{\textrm{ave}}$ can be computed. Here, $Y_{\textrm{ave}}$ corresponds to the average of the integral of Eq. (8) over the angular interval $[\alpha _{\textrm{min}}, \, \alpha _{\textrm{max}}]$, such that:

10 $$\begin{aligned} Y_{\textrm{ave}} (E_0) = \frac{1}{\alpha _{\textrm{max}} - \alpha _{\textrm{min}}} \int _{\alpha _{\textrm{min}}}^{\alpha _{\textrm{max}}} Y(E_0,\ \alpha ) \ d\alpha \end{aligned}$$

**Fig. 9:**
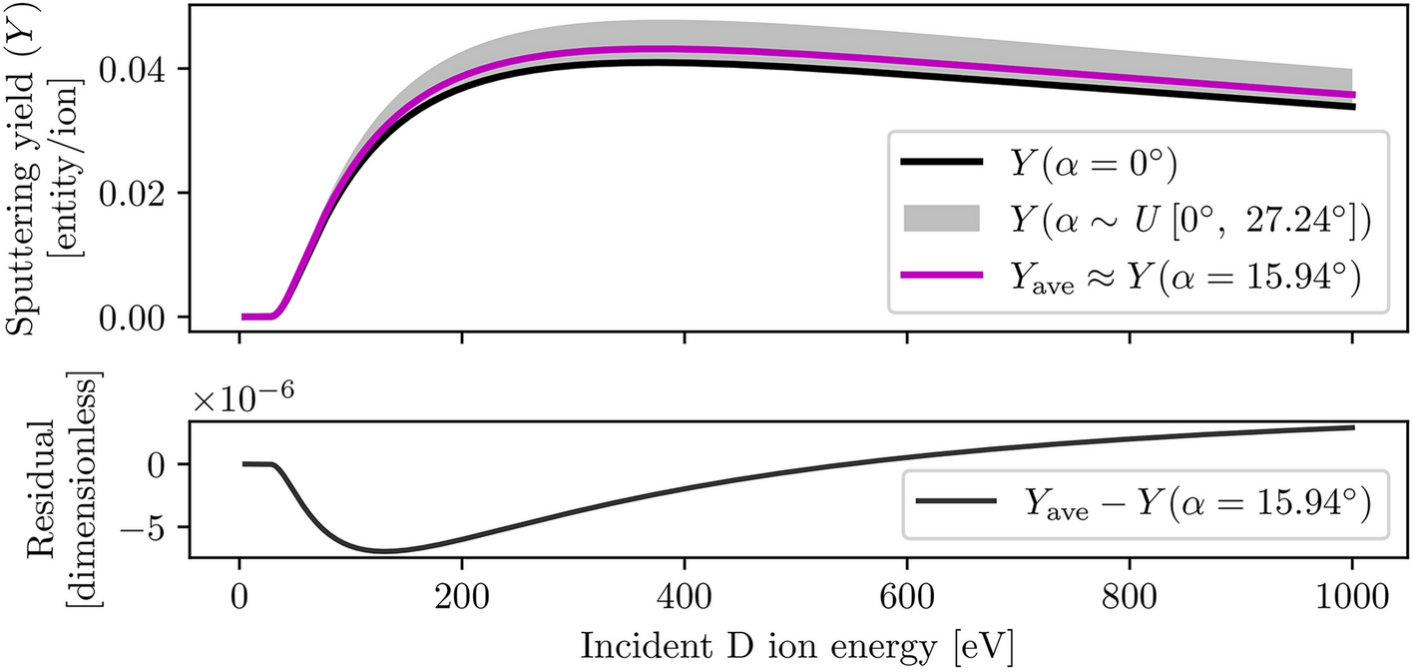
Comparison between the sputtering yield at normal ion incidence $Y(\alpha =0^{\circ })$ (black line), the valid region of sputtering yields $Y(\alpha )$ (grey region) for a uniform ion angular distribution simulated by the MD model, and the integrated average sputtering yield $Y_{\textrm{ave}}$ (magenta line) over the valid region. An average ion incidence angle $\alpha _{\textrm{ave}}$ was obtained by minimisation of $| Y_{\textrm{ave}} - Y(\alpha ) |$ within the applicable range of incidence angles, such that $Y(\alpha _{\textrm{ave}})$ reasonably approximates $Y_{\textrm{ave}}$; see the lower black curve for the minimisation residual at the final $\alpha _{\textrm{ave}}$ value.

To obtain $\alpha _{\textrm{ave}}$, the value of $\alpha$ at which Eq. (8) approximately evaluates to $Y_{\textrm{ave}}$ needs to be computed. This can be accomplished by minimising the function $f(E_0, \alpha ) = | Y_{\textrm{ave}} - Y(E_0, \alpha ) |$ over the previously specified interval $[\alpha _{\textrm{min}}, \, \alpha _{\textrm{max}}]$. Using sample Bohdasnky parameters $Q=0.181$ atoms/ion and $E_{\textrm{th}}=26.2$ eV representing the experimentally measured sputtering yield of mixed beryllium targets by deuterium ions at room temperature^26^, the minimisation yields the value $\alpha _{\textrm{ave}} = 15.94^{\circ }$ estimated to two decimal places, as illustrated in Fig. 9. When substituted into Eq. (8), the resulting curve represents a reasonable approximation of the expected average sputtering yield for our bombarding ion angular distribution, and as such, all data fitted to Eq. (8) reasonably assumes a fixed parameter $\alpha = 15.94^{\circ }$ throughout this work. It should be noted that the minimised $\alpha _{\textrm{ave}}$ is not sensitive to the choice of Bohdansky parameters (independent of *Q*, $8\text {eV} \le E_{\textrm{th}} \le 98\text {eV}$), and should therefore be valid for any reasonable choice of *Q* and $E_{\textrm{th}}$ for beryllium sputtering by hydrogenic ions.

## Supplementary Information

Supplementary Information 1.

Supplementary Information 2.

## Data Availability

The datasets used and/or analysed during the current study are available from the corresponding author on reasonable request.
