# Supplementary material for: A molecular dynamics study of the sputtering processes of beryllium species by hydrogenic plasma
Source: Sci Rep. 2025 Apr 29;15:15068. doi: 10.1038/s41598-025-98065-1 (PMC12041515; doi:10.1038/s41598-025-98065-1)
Supplement: Supplementary file 1 — Supplementary Information 1. [file 41598_2025_98065_MOESM1_ESM.pdf]

A molecular dynamics study of the sputtering processes of beryllium species by hydrogenic plasma - Supplementary information

Alexander Liptak[1,2,*], Kerry D Lawson[3], and Mohammad I Hasan[1,*]

[1]Department of Electrical Engineering and Electronics, University of Liverpool, Liverpool, L69 3GJ, United Kingdom
[2]Diamond Light Source Ltd, Harwell Science and Innovation Campus, Didcot, OX11 0DE, United Kingdom
[3]UKAEA (UK Atomic Energy Authority), Culham Campus, Abingdon, OX14 3DB, United Kingdom
[*]Alexander.Liptak@liverpool.ac.uk, mihasan@liverpool.ac.uk

Supplementary media: sub-threshold sputtering

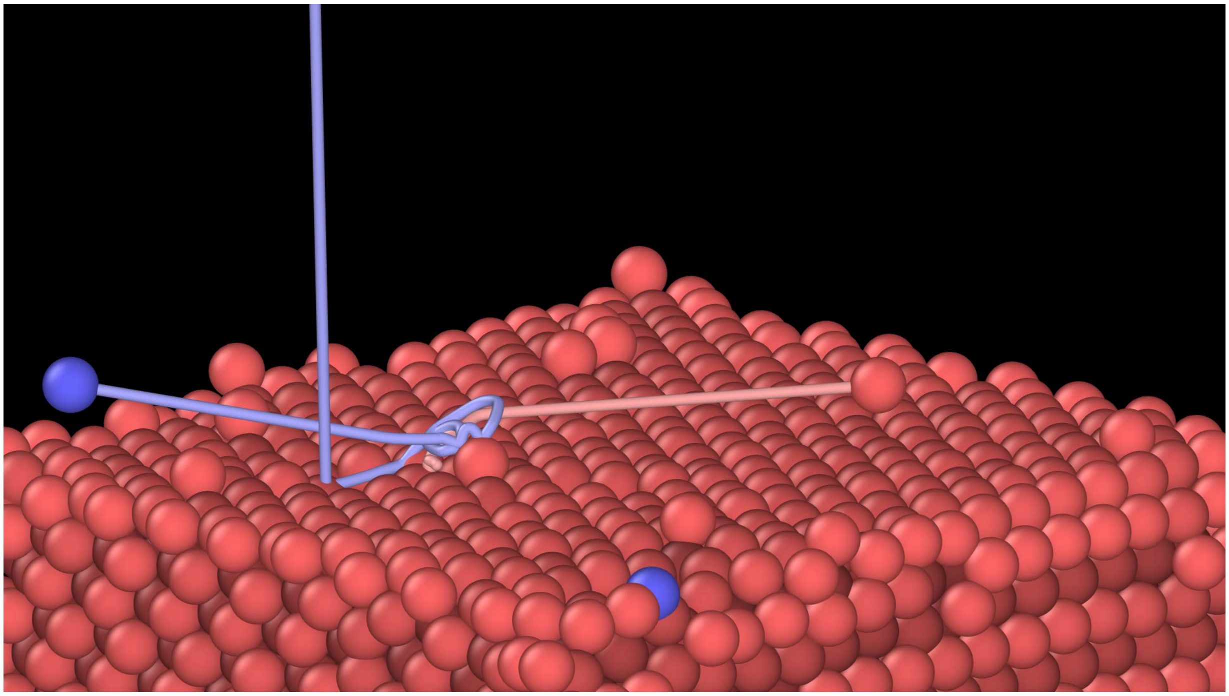

Figure 1. Sub-threshold sputtering of atomic beryllium resulting from the bombardment of a $T_{\mathrm{Be}} = 300\,\mathrm{K}$ beryllium lattice (red) by a $T_{\mathrm{T}} = 14\,\mathrm{eV}$ tritium ion (blue). While the sputtering threshold energy defines the minimum energy for physical sputtering, chemically-assisted sputtering processes such as swift chemical sputtering (SCS) can still occur at sub-threshold energies. Although the presented model does not simulate interatomic bonding, SCS-like sputtering arising directly from the attractive force of the interatomic potential has been demonstrated here on BeD and BeT trimers. This video illustrates such an event: the sub-threshold sputtering of a BeT dimer, which promptly disassociates into individual beryllium and tritium atoms due to interactions with nearby beryllium surface atoms; as sputtering events are only classified once the sputtered entities are no longer under the influence of the surface, this event is classified as the sputtering of atomic beryllium, although occurring at a sub-threshold tritium bombardment energy.

Supplementary tables: summary of bombardments and sputtering events

The data presented in this study reflects the analysis of 23,766 simulated non-cumulative bombardments of beryllium lattices with surface temperatures up to 1100 K by deuterium and tritium ions with energies up to 1 keV. The following tables, grouped by lattice surface temperature and bombarding ion isotope, and split by a horizontal line into the standard (<= 150 eV) and extended (> 150 eV) bombardment energy regimes, include information about:

- The total number of bombardments N_{total} at each ion energy T_D or T_T
- Classification of bombarding ion trajectories into reflected (N_{refl}, number of bombardments where the bombarding ion reflects from the top surface), sorbed (N_{sorb}, number of bombardments where the bombarding ion adsorbed onto the top surface or was absorbed into the bulk of the lattice), and transmitted (N_{trans}, number of bombardments where the bombarding ion was absorbed, passed through the bulk of the lattice, and desorbed from the bottom surface), such that $N_{refl} + N_{refl} + N_{trans} = N_{total}$
- The sputtering yield of atomic beryllium Y_{Be}, the beryllium dimer Y_{Be_2}, and the beryllium hydride dimers Y_{BeD} or Y_{BeT}, where the sputtering yields resulting from reflection of the bombarding ion are included in parentheses for each sputtered species.

T_D [eV]	N_{total}	N_{refl}	N_{sorb}	N_{trans}	Y_{Be} (by refl.)	Y_{Be_2} (by refl.)	Y_{BeD} (by refl.)
5	100	42	58	0	0 (0)	0 (0)	0 (0)
10	100	35	65	0	0 (0)	0 (0)	0 (0)
11	100	30	70	0	0 (0)	0 (0)	0 (0)
12	100	36	64	0	0 (0)	0 (0)	0 (0)
13	100	41	59	0	0 (0)	0 (0)	0 (0)
14	100	28	72	0	0 (0)	0 (0)	0 (0)
15	180	78	102	0	0 (0)	0 (0)	0 (0)
16	100	45	55	0	0 (0)	0 (0)	0 (0)
17	100	49	51	0	0 (0)	0 (0)	0 (0)
18	100	37	63	0	0 (0)	0 (0)	1 (1)
19	100	39	60	1	0 (0)	0 (0)	0 (0)
20	100	43	56	1	0 (0)	0 (0)	0 (0)
21	100	37	62	1	1 (0)	0 (0)	0 (0)
22	100	50	49	1	0 (0)	0 (0)	0 (0)
23	100	43	57	0	1 (0)	0 (0)	0 (0)
24	100	45	54	1	2 (0)	0 (0)	0 (0)
25	100	40	58	2	3 (0)	0 (0)	1 (1)
30	100	36	64	0	2 (0)	0 (0)	0 (0)
35	100	51	49	0	7 (3)	0 (0)	0 (0)
40	283	101	180	2	11 (1)	0 (0)	0 (0)
45	300	108	191	1	21 (4)	0 (0)	0 (0)
50	300	123	176	1	32 (4)	1 (0)	1 (1)
60	164	59	105	0	22 (7)	0 (0)	0 (0)
70	156	53	103	0	23 (9)	0 (0)	2 (2)
80	164	56	108	0	21 (3)	1 (0)	0 (0)
90	164	57	107	0	28 (11)	0 (0)	0 (0)
100	167	59	108	0	43 (10)	0 (0)	1 (1)
150	100	17	83	0	25 (2)	1 (0)	0 (0)
200	174	52	118	4	54 (18)	0 (0)	0 (0)
300	162	31	124	7	72 (12)	1 (0)	0 (0)
400	172	43	119	10	89 (28)	5 (3)	0 (0)
500	174	31	118	25	118 (35)	4 (0)	1 (1)
600	187	43	103	41	104 (28)	4 (4)	0 (0)
700	200	37	105	58	101 (25)	3 (2)	0 (0)
800	200	29	83	88	85 (27)	6 (3)	0 (0)
900	200	34	72	94	93 (36)	3 (1)	1 (1)
1000	200	35	72	93	87 (32)	3 (2)	0 (0)

Table 1. Bombardment statistics, sputtering yield and preferential sputtering data of atomic Be, the Be_2 and BeD dimers from a T_{Be} = 300 K beryllium lattice by deuterium ions.

T_D [eV]	N_{total}	N_{refl}	N_{sorb}	N_{trans}	Y_{Be} (by refl.)	Y_{Be_2} (by refl.)	Y_{BeD} (by refl.)
5	100	42	58	0	0 (0)	0 (0)	0 (0)
10	90	31	59	0	0 (0)	0 (0)	0 (0)
11	100	37	63	0	0 (0)	0 (0)	0 (0)
12	100	33	67	0	0 (0)	0 (0)	0 (0)
13	100	37	63	0	0 (0)	0 (0)	0 (0)
14	100	42	58	0	0 (0)	0 (0)	1 (1)
15	100	38	62	0	0 (0)	0 (0)	0 (0)
16	100	38	62	0	0 (0)	0 (0)	0 (0)
17	100	34	66	0	0 (0)	0 (0)	0 (0)
18	100	38	62	0	1 (1)	0 (0)	0 (0)
19	100	37	63	0	1 (0)	0 (0)	0 (0)
20	99	41	58	0	0 (0)	0 (0)	3 (3)
21	100	38	62	0	0 (0)	0 (0)	0 (0)
22	100	40	60	0	4 (1)	0 (0)	0 (0)
23	100	47	53	0	2 (0)	0 (0)	0 (0)
24	100	43	57	0	1 (0)	0 (0)	0 (0)
25	100	36	64	0	1 (0)	0 (0)	0 (0)
30	100	39	60	1	10 (0)	0 (0)	0 (0)
35	100	37	62	1	10 (1)	0 (0)	0 (0)
40	100	40	60	0	9 (2)	0 (0)	0 (0)
45	100	45	55	0	7 (1)	0 (0)	0 (0)
50	100	41	59	0	15 (5)	0 (0)	1 (1)
60	100	38	62	0	10 (3)	0 (0)	2 (2)
70	100	43	57	0	19 (7)	1 (0)	0 (0)
80	100	30	70	0	18 (4)	0 (0)	0 (0)
90	100	38	62	0	17 (6)	0 (0)	2 (2)
100	100	24	75	1	32 (10)	0 (0)	0 (0)
150	100	33	66	1	42 (11)	0 (0)	0 (0)
200	100	25	73	2	29 (10)	6 (6)	1 (1)
300	100	26	68	6	36 (12)	0 (0)	0 (0)
400	100	27	68	5	61 (20)	3 (3)	0 (0)
500	100	23	64	13	60 (15)	1 (1)	0 (0)
600	100	22	58	20	54 (30)	17 (8)	0 (0)
700	100	16	52	32	28 (7)	9 (3)	0 (0)
800	100	20	49	31	60 (27)	2 (0)	0 (0)
900	100	15	42	43	25 (2)	1 (1)	0 (0)
1000	100	13	34	53	47 (13)	9 (3)	0 (0)

Table 2. Bombardment statistics, sputtering yield and preferential sputtering data of atomic Be, the Be_2 and BeD dimers from a T_{Be} = 500 K beryllium lattice by deuterium ions.

T_D [eV]	N_{total}	N_{refl}	N_{sorb}	N_{trans}	Y_{Be} (by refl.)	Y_{Be_2} (by refl.)	Y_{BeD} (by refl.)
5	100	43	57	0	0 (0)	0 (0)	0 (0)
10	89	29	60	0	0 (0)	0 (0)	0 (0)
11	100	29	71	0	0 (0)	0 (0)	0 (0)
12	100	38	62	0	0 (0)	0 (0)	0 (0)
13	100	37	63	0	0 (0)	0 (0)	0 (0)
14	100	32	68	0	0 (0)	0 (0)	0 (0)
15	100	44	56	0	0 (0)	0 (0)	0 (0)
16	100	44	56	0	0 (0)	0 (0)	0 (0)
17	100	40	60	0	0 (0)	0 (0)	1 (1)
18	100	50	50	0	1 (0)	0 (0)	1 (1)
19	100	38	62	0	1 (0)	0 (0)	0 (0)
20	95	38	57	0	0 (0)	0 (0)	1 (1)
21	100	34	66	0	0 (0)	0 (0)	0 (0)
22	100	38	62	0	0 (0)	0 (0)	0 (0)
23	100	46	54	0	0 (0)	0 (0)	0 (0)
24	100	46	54	0	1 (0)	0 (0)	1 (1)
25	100	38	62	0	1 (0)	0 (0)	0 (0)
30	96	37	59	0	6 (1)	0 (0)	1 (1)
35	100	35	65	0	8 (1)	0 (0)	0 (0)
40	95	29	66	0	15 (1)	0 (0)	1 (1)
45	100	40	60	0	5 (0)	0 (0)	1 (1)
50	100	43	57	0	9 (1)	0 (0)	2 (2)
60	98	30	68	0	12 (2)	1 (0)	0 (0)
70	100	37	63	0	19 (5)	1 (0)	0 (0)
80	100	24	76	0	26 (5)	0 (0)	2 (2)
90	100	35	65	0	18 (9)	0 (0)	1 (1)
100	100	26	74	0	20 (5)	0 (0)	0 (0)
150	100	36	64	0	36 (10)	0 (0)	1 (1)
200	100	22	77	1	38 (12)	4 (3)	0 (0)
300	100	20	77	3	51 (11)	2 (0)	0 (0)
400	100	25	71	4	39 (15)	6 (6)	0 (0)
500	100	19	70	11	59 (25)	11 (2)	0 (0)
600	100	18	50	32	46 (15)	0 (0)	0 (0)
700	100	16	49	35	61 (17)	2 (0)	0 (0)
800	100	22	48	30	43 (7)	4 (3)	0 (0)
900	100	18	37	45	44 (14)	0 (0)	0 (0)
1000	100	13	30	57	53 (11)	5 (1)	0 (0)

Table 3. Bombardment statistics, sputtering yield and preferential sputtering data of atomic Be, the Be_2 and BeD dimers from a T_{Be} = 700 K beryllium lattice by deuterium ions.

T_D [eV]	N_{total}	N_{refl}	N_{sorb}	N_{trans}	Y_{Be} (by refl.)	Y_{Be_2} (by refl.)	Y_{BeD} (by refl.)
5	100	40	60	0	0 (0)	0 (0)	0 (0)
10	86	30	56	0	0 (0)	0 (0)	0 (0)
11	100	38	62	0	0 (0)	0 (0)	0 (0)
12	100	40	60	0	0 (0)	0 (0)	0 (0)
13	100	50	50	0	0 (0)	0 (0)	0 (0)
14	100	28	72	0	0 (0)	0 (0)	0 (0)
15	100	31	69	0	1 (0)	0 (0)	0 (0)
16	100	40	60	0	0 (0)	0 (0)	0 (0)
17	100	34	66	0	1 (0)	0 (0)	0 (0)
18	100	39	61	0	1 (0)	0 (0)	2 (2)
19	100	44	56	0	1 (0)	0 (0)	1 (1)
20	89	33	56	0	1 (0)	0 (0)	0 (0)
21	100	34	66	0	3 (0)	0 (0)	1 (1)
22	100	33	67	0	0 (0)	0 (0)	0 (0)
23	100	47	53	0	1 (0)	0 (0)	0 (0)
24	100	49	51	0	2 (0)	0 (0)	0 (0)
25	100	41	59	0	1 (0)	0 (0)	0 (0)
30	94	30	64	0	3 (0)	0 (0)	0 (0)
35	100	46	54	0	1 (0)	0 (0)	0 (0)
40	96	35	61	0	8 (2)	0 (0)	0 (0)
45	100	29	71	0	13 (1)	0 (0)	0 (0)
50	100	38	62	0	11 (2)	1 (0)	1 (1)
60	87	22	65	0	12 (3)	0 (0)	0 (0)
70	100	34	66	0	22 (8)	0 (0)	0 (0)
80	200	72	28	0	43 (13)	0 (0)	0 (0)
90	100	36	64	0	9 (6)	1 (1)	0 (0)
100	100	28	72	0	15 (6)	0 (0)	1 (1)
150	100	28	72	0	29 (8)	0 (0)	0 (0)
200	100	26	73	1	35 (8)	2 (1)	0 (0)
300	100	19	77	4	45 (14)	2 (0)	0 (0)
400	100	25	68	7	58 (15)	4 (3)	0 (0)
500	100	23	57	20	59 (18)	0 (0)	0 (0)
600	100	19	65	16	44 (12)	3 (0)	0 (0)
700	100	18	54	28	40 (12)	3 (1)	0 (0)
800	100	18	53	29	48 (10)	1 (1)	0 (0)
900	100	13	41	46	44 (8)	1 (1)	0 (0)
1000	100	14	31	55	53 (5)	1 (0)	0 (0)

Table 4. Bombardment statistics, sputtering yield and preferential sputtering data of atomic Be, the Be_2 and BeD dimers from a T_{Be} = 900 K beryllium lattice by deuterium ions.

T_D [eV]	N_{total}	N_{refl}	N_{sorb}	N_{trans}	Y_{Be} (by refl.)	Y_{Be_2} (by refl.)	Y_{BeD} (by refl.)
5	100	41	59	0	0 (0)	0 (0)	0 (0)
10	100	39	61	0	0 (0)	0 (0)	2 (2)
11	100	26	74	0	0 (0)	0 (0)	0 (0)
12	100	31	69	0	0 (0)	0 (0)	0 (0)
13	100	36	64	0	0 (0)	0 (0)	0 (0)
14	100	33	67	0	0 (0)	0 (0)	0 (0)
15	100	35	65	0	0 (0)	0 (0)	1 (1)
16	100	42	58	0	0 (0)	0 (0)	0 (0)
17	100	37	63	0	0 (0)	0 (0)	0 (0)
18	100	35	65	0	1 (0)	0 (0)	1 (1)
19	100	40	60	0	1 (0)	0 (0)	0 (0)
20	100	36	64	0	3 (1)	0 (0)	1 (1)
21	100	34	66	0	0 (0)	0 (0)	0 (0)
22	100	44	56	0	2 (0)	0 (0)	0 (0)
23	100	36	64	0	0 (0)	0 (0)	2 (2)
24	100	35	65	0	5 (0)	0 (0)	0 (0)
25	100	39	61	0	3 (0)	0 (0)	2 (2)
30	100	43	57	0	1 (0)	0 (0)	1 (1)
35	100	38	62	0	5 (1)	0 (0)	0 (0)
40	100	39	61	0	8 (1)	0 (0)	1 (1)
45	100	34	66	0	11 (3)	0 (0)	1 (1)
50	100	41	59	0	10 (1)	0 (0)	0 (0)
60	100	33	67	0	13 (4)	0 (0)	0 (0)
70	100	30	70	0	12 (2)	0 (0)	0 (0)
80	100	34	66	0	16 (7)	0 (0)	0 (0)
90	100	32	68	0	21 (8)	0 (0)	1 (1)
100	100	33	67	0	27 (7)	0 (0)	0 (0)
150	100	28	70	2	33 (13)	0 (0)	0 (0)
200	100	34	66	0	45 (16)	0 (0)	0 (0)
300	100	26	71	3	47 (18)	2 (2)	0 (0)
400	100	23	70	7	69 (11)	6 (3)	0 (0)
500	100	24	63	13	57 (17)	1 (0)	1 (1)
600	100	24	55	21	49 (15)	9 (7)	0 (0)
700	100	24	54	22	61 (15)	6 (0)	0 (0)
800	100	30	31	39	50 (22)	12 (5)	0 (0)
900	100	24	36	40	51 (12)	2 (1)	1 (1)
1000	100	15	30	55	32 (9)	4 (2)	0 (0)

Table 5. Bombardment statistics, sputtering yield and preferential sputtering data of atomic Be, the Be_2 and BeD dimers from a T_{Be} = 1100 K beryllium lattice by deuterium ions.

T_T [eV]	N_{total}	N_{refl}	N_{sorb}	N_{trans}	Y_{Be} (by refl.)		Y_{Be_2} (by refl.)		Y_{BeT} (by refl.)	
5	100	30	70	0	0	(0)	0	(0)	0	(0)
10	100	21	79	0	0	(0)	0	(0)	0	(0)
11	100	28	72	0	0	(0)	0	(0)	0	(0)
12	100	27	73	0	0	(0)	0	(0)	0	(0)
13	100	37	63	0	0	(0)	0	(0)	0	(0)
14	100	26	74	0	1	(1)	0	(0)	0	(0)
15	100	31	68	1	0	(0)	0	(0)	1	(1)
16	100	26	74	0	0	(0)	0	(0)	0	(0)
17	100	25	75	0	0	(0)	0	(0)	0	(0)
18	100	28	72	0	0	(0)	0	(0)	1	(1)
19	100	31	68	1	0	(0)	0	(0)	0	(0)
20	100	27	73	0	0	(0)	0	(0)	0	(0)
21	100	25	75	0	0	(0)	0	(0)	0	(0)
22	100	26	74	0	1	(0)	0	(0)	0	(0)
23	100	26	73	1	1	(0)	0	(0)	1	(1)
24	100	32	68	0	0	(0)	0	(0)	0	(0)
25	100	32	66	2	1	(0)	0	(0)	0	(0)
30	100	27	72	1	1	(0)	0	(0)	0	(0)
40	100	31	68	1	6	(0)	0	(0)	0	(0)
50	100	35	65	0	6	(2)	0	(0)	0	(0)
60	100	23	77	0	7	(1)	0	(0)	0	(0)
70	100	21	79	0	11	(1)	0	(0)	0	(0)
80	100	22	78	0	20	(4)	0	(0)	0	(0)
90	100	25	75	0	23	(3)	1	(0)	1	(1)
100	105	26	79	0	18	(2)	0	(0)	1	(1)
150	100	18	80	2	30	(5)	0	(0)	0	(0)
200	100	20	80	0	40	(6)	1	(1)	0	(0)
300	100	15	79	6	41	(9)	0	(0)	0	(0)
400	100	21	76	3	67	(16)	0	(0)	0	(0)
500	100	13	76	11	37	(11)	1	(0)	0	(0)
600	100	19	64	17	73	(29)	1	(0)	0	(0)
700	100	13	72	15	67	(10)	2	(1)	0	(0)
800	100	11	62	27	71	(18)	2	(1)	0	(0)
900	100	11	49	40	46	(10)	3	(0)	0	(0)
1000	100	17	39	44	62	(17)	3	(1)	0	(0)

Table 6. Bombardment statistics, sputtering yield and preferential sputtering data of atomic Be, the Be_2 and BeT dimers from a T_{Be} = 300 K beryllium lattice by tritium ions.
