# Supplementary figures and images for: A molecular dynamics study of the sputtering processes of beryllium species by hydrogenic plasma
Source: Sci Rep. 2025 Apr 29;15:15068. doi: 10.1038/s41598-025-98065-1 (PMC12041515; doi:10.1038/s41598-025-98065-1)

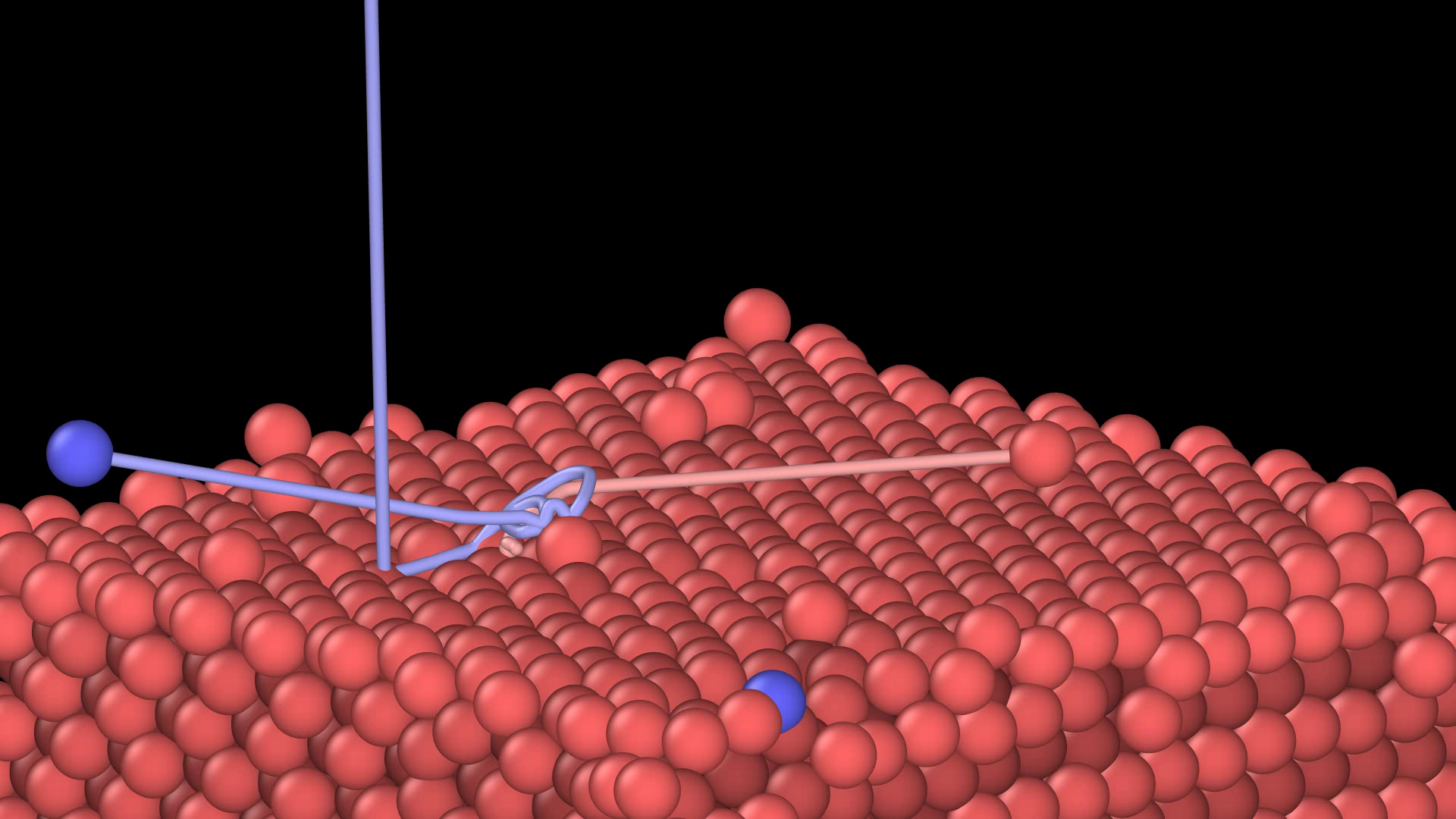

Supplement: Supplementary file 2 — Supplementary Information 2. [file 41598_2025_98065_MOESM2_ESM.zip › sput_BeD_as_Be.png]
